# Is depression the contraindication of anterior cervical decompression and fusion for cervical spondylosis?

**DOI:** 10.3389/fendo.2022.1031616

**Published:** 2022-09-30

**Authors:** Xiaolu Chen, Xiao Li, Yu Gan, Ying Lu, Yu Tian, Yixiao Fu, Hanjie Yang, Ke Liu, Yinlian Pan, Xing Du

**Affiliations:** ^1^ Department of Psychiatry, The First Branch, The First Affiliated Hospital of Chongqing Medical University, Chongqing, China; ^2^ Department of Psychiatry, The First Affiliated Hospital of Chongqing Medical University, Chongqing, China; ^3^ Department of Psychiatry, Chongqing Eleventh People’s Hospital, Chongqing, China; ^4^ Department of the First Clinical Medicine, Chongqing Medical University, Chongqing, China; ^5^ Department of Neurology, The Thirteenth People’s Hospital of Chongqing, Chongqing, China; ^6^ Department of Emergency, The Second Affiliated Hospital of Chongqing Medical University, Chongqing, China; ^7^ Department of Medical Oncology, The First Affiliated Hospital of Hainan Medical University, Haikou, China; ^8^ Department of Orthopedics, The First Affiliated Hospital of Chongqing Medical University, Chongqing, China

**Keywords:** depression, anterior cervical decompression and fusion, cervical spondylosis, clinical efficacy, safety

## Abstract

**Objective:**

To evaluate whether depression is the contraindication of anterior cervical decompression and fusion (ACDF) for cervical spondylosis.

**Material and methods:**

Patients with single-segment cervical spondylosis who underwent ACDF from January 2015 to December 2018 in our department were retrospectively included in this study and divided into two groups. Patients who were diagnosed of depression and prescribed with antidepressant drugs for at least 6 months before surgery were included in the intervention group. Patients without depression were included in the control group. The Beck Depression Inventory (BDI) score was used to evaluate the severity of depression. Visual Analogue Scale (VAS) score, Japanese Orthopeadic Association (JOA) score, Neck Disability Index (NDI), and the 36-Item Short-Form Health Survey (SF-36) were recorded as indexes to assess the pain, cervical spine function, degree of cervical spine injury, and life quality, respectively. The operative time, operative blood loss, hospital stay and complications were also recorded and compared.

**Results:**

A total of 117 patients were included in this study, involving 32 patients in the intervention group and 85 patients in the control group. No significant differences were found in operative time, operative blood loss, hospital stay and complications between the two groups (P>0.05). The BDI score, VAS score, JOA score, NDI, SF-36 physical component score (SF-36 PCS) and SF-36 mental component score (SF-36 MCS) were all significantly improved at last follow-up in both the two groups. The intervention group showed higher BDI score and SF-36 MCS than the control group at both preoperative and the last follow-up (P<0.05), and the improvements of BDI score and SF-36 MCS were also higher in the intervention group (P<0.05). Although the intervention group showed higher VAS score, NDI, SF-36 PCS and lower JOA score at preoperative and last follow-up, respectively (P<0.05), there were no significant differences in the improvements of these indexes between the two group (P>0.05).

**Conclusions:**

Depression is not the contraindication of ACDF for cervical spondylosis. Depression patients who received preoperative antidepressants can achieve similar improvement of clinical symptoms from ACDF with non-depression patients.

## Introduction

Anterior cervical decompression and fusion (ACDF) is a common surgical method and has been regarded as the the gold standard treatment for cervical spondylosis (1). However, in recent years, clinical reports showed that the improvements of pain and life quality were not as satisfactory as preoperative expected in some cervical spondylosis patients undergoing ACDF (2, 3). These variable surgical outcomes prompted us to seek for the preoperative factors that may affect the clinical efficacy of ACDF.

Cervical spondylosis patients are often complicated with psychological disorders (4, 5). It is reported that more than 30% of cervical spondylosis patients suffer from depression or anxiety (6). Prior studies have identified that preoperative psychological disorders, such as anxiety and depression, are negatively correlated with the improvement of functional outcomes and life quality in cervical spondylosis patients undergoing cervical spine surgery (7, 8). It was reported that in the US patients undergoing ACDF, pre-surgical clinical depression predicts post-surgical acute or chronic pain, a slightly prolonged length of hospital stay and the presence of any complication (9). Harris et al. found that patients with preoperative diagnoses of depression or anxiety had a greater likelihood of adverse outcomes, increased opioid consumption, and increased cumulative health care payments after ACDF compared with patients without depression or anxiety (10). Since depression had a negative impact on the efficacy of ACDF surgery, antidepressant treatment may improve the efficacy of ACDF for depression patients.

However, little data so far had assessed the efficacy and safety of antidepressant medications in ACDF. Elsamadicy et al. found that preoperative antidepressant treatment could significantly improve postoperative pain and dysfunction in patients with depression after cervical surgery (11). However, Sayadipour et al. concluded that preoperative antidepressants treatment increased the total cost of medical treatment and prolonged the hospital stay in patients with depression undergoing elective lumbar surgery (12). Moreover, it was reported that the use of antidepressants may lead to an increased risk of abnormal bleeding in orthopedic surgery (13). Therefore, the safety and efficacy of antidepressant therapy before ACDF in patients with depression are remain controversial.

Therefore, we conducted this retrospective study to compare the pain relief and functional outcomes improvement of ACDF between the normal patients and depression patients to evaluate whether depression is the contraindication of ACDF. The main hypothesis was that, depression is not the contraindication of ACDF for cervical spondylosis, and depression patients who received preoperative standard antidepressants can achieve similar improvement of clinical symptoms from ACDF with non-depression patients.

## Materials and methods

This study was approved by the Ethics Committee of The First Affiliated hospital of Chongqing Medical University (No: 2017-98). All of the participants provided their written informed consent to participate in this study before their data were stored in the hospital database and used for research purposes. This work was reported in line with the STROCSS criteria (14).

### Patients selection

From January 2015 to December 2018, patients who underwent ACDF for cervical spondylosis were retrospectively included in our study.

Inclusion criteria: (1) patients with symptoms and signs of cervical spondylotic radiculopathy (unilateral/bilateral upper limb pain, numbness or weakness, negative pathological signs); (2) preoperative MRI of the cervical spine revealed compression of a single segment of the nerve root; (3) patients with progressive neurological deficit; (4) patients undergoing conservative therapy for at least 3 months without improvement; (5) patients without depression (control group) or was diagnosed as depression by a psychiatrist and took antidepressants for at least 6 months before surgery (intervention group).

Exclusion criteria: (1) patients who took drugs that may affect the antidepressant medications efficacy assessment within 1 month before surgery (e.g., calcium channel blockers, corticosteroids, methotrexate, a vitamin K antagonist, or an antiplatelet medication; (2) patients with a previous history of spine surgery; (3) patients with recurrent cervical spondylosis or re-hospitalization due to a revision cervical surgery.

### Preoperative management

All patients received X-ray in cervical flexion and extension position, CT and MRI. Surgery was conducted after the patients’ basic diseases such as diabetes, coronary heart disease and hypertension, were controlled and stabilized.

### Surgical method

All patients underwent ACDF *via* the anterior Smith–Robinson approach. The procedures were similar with previous article (15). Two types of cages were used: PEEK cage (Medtronic, USA) and nHA-PA66 cages (Guona, China).

### Postoperative management

Prophylactic use of antibiotics for the first 3 days after surgery. Incision drainage was removed when drainage volume was less than 10 ml/d, and then an X-ray examination was checked. A neck collar fixation was applied for postoperative 3 months. X-ray and MRI (if necessary) were followed up to 1, 3, 6, 12, 24 months postoperatively.

### Assessment measures

Patients basic demographics: age, gender, duration of symptoms, body mass index (BMI), employment status, smoking history, radiculopathy laterality, and disease level.

Clinical outcomes: (1) Surgery-related outcomes: operative time, operative blood loss, hospital stay and complications. (2) Pain: visual analogue scale (VAS) score. (3) Depression: the Beck Depression Inventory (BDI) score. (4) Cervical spine function: Japanese Orthopeadic Association (JOA) score and neck disability index (NDI). (5) Life quality: the 36-Item Short-Form Health Survey (SF-36).

### Statistical analysis

Matched *t* test was used to compare VAS score, BDI score and SF-36 score between preoperative and postoperative period. Independent samples *t* test and Chi-square test were used for the comparison of quantitative data (e.g. operative time, operative blood loss) and disordered qualitative data (e.g. postoperative complications) between the intervention group and the control group, respectively. SPSS 17.0.1 (Chicago, USA) was used for statistical analysis. P<0.05 was considered to be the significant difference.

## Results

**Figure 1:**
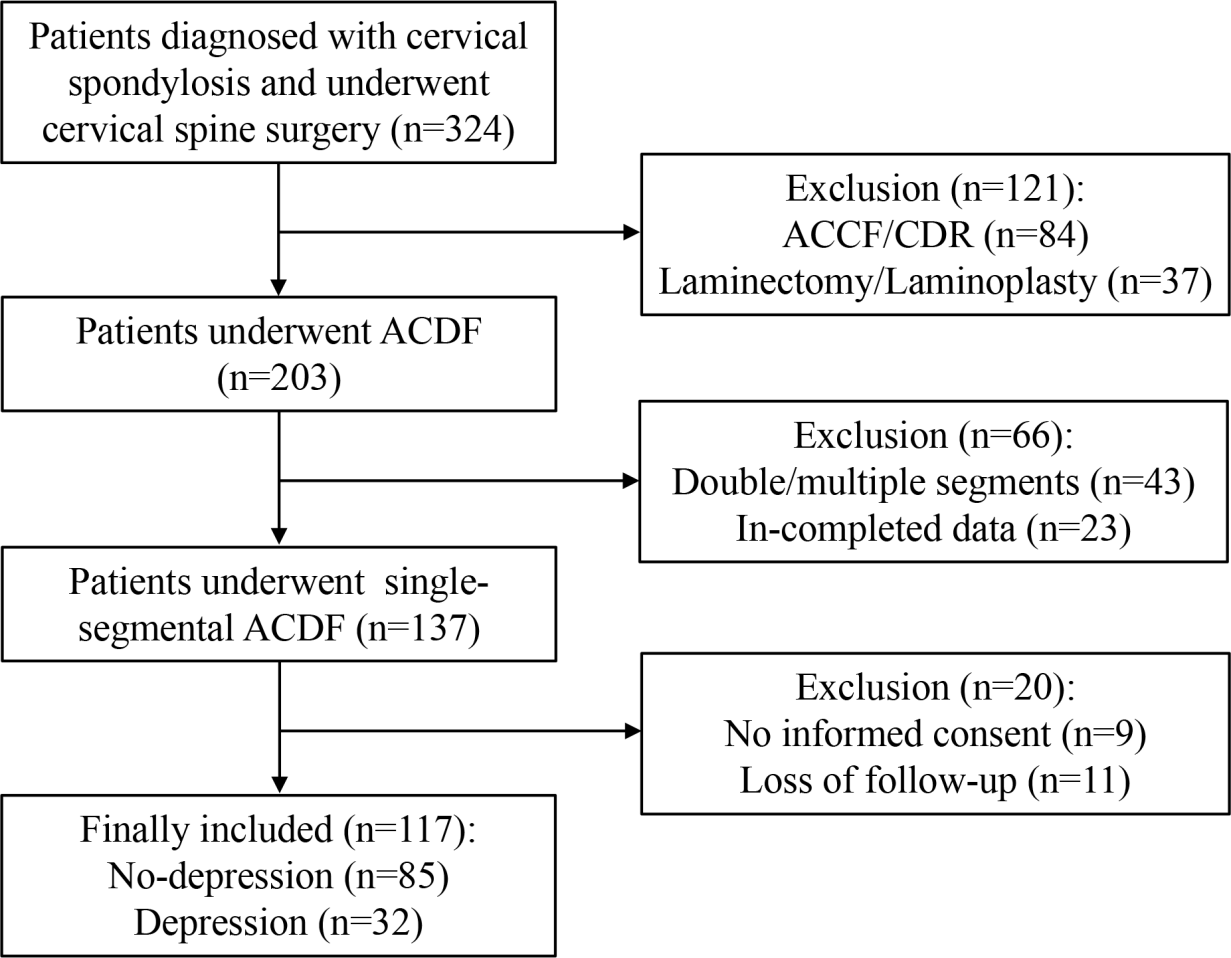
Flow chart of the inclusion and exclusion of patients.

A total of 117 patients were included in this study, involving 32 patients in the intervention group and 85 patients in the control group (**Figure 1**). No significant differences were found in age (P=0.373), gender (P=0.061), duration of symptoms (P=0.243), BMI (P=0.317), employment status (P=0.385), smoking history (P=0.342), radiculopathy laterality (P=0.542), disease level (P=0.507), and follow-up time (P=0.373) between the two groups. (**Table 1**)

**Table 1:** Comparison of clinical basic characteristics between the two groups.

	Control group (N = 85)	Intervention group (N = 32)	*P* value
Age, yr	60.3 ± 10.5	58.5 ± 9.4	0.373
Gender, n			0.061
Male	18	12	
Female	67	20	
Duration of symptoms, month	12.5 ± 4.1	13.6 ± 5.5	0.243
BMI, kg/m^2^	28.6 ± 6.7	30.1 ± 8.4	0.317
Employment status, n			0.385
Working/studying	15	4	
Unemployed or retiree	70	28	
Smoking history, n			0.342
Yes	19	8	
No	66	24	
Radiculopathy laterality, n			0.542
Bilateral	25	9	
Unilateral	60	23	
Disease Level, n			0.507
C3/4	14	5	
C4/5	34	13	
C5/6	27	10	
C6/7	10	4	

In both the two groups, the BDI score, VAS, JOA score, NDI, SF-36 physical component score (SF-36 PCS) and SF-36 mental component score (SF-36 MCS) were all significantly improved at last follow-up compared with preoperative (all P<0.05). The intervention group showed higher BDI score (both P<0.001) and higher SF-36 MCS (P=0.002 and P<0.001, respectively) than the control group at both preoperative and the last follow-up, and the changes of BDI score (P=0.014) and SF-36 MCS (P=0.031) were also higher in the intervention group. Although the intervention group showed higher VAS (P=0.020 and P=0.011, respectively), lower JOA score (P=0.014 and P<0.001, respectively), higher NDI (both P<0.001), and higher SF-36 PCS (P=0.002 and P<0.001, respectively) at preoperative and last follow-up, there were no significant differences in the changes of these indexes between the two group (P=0.087, 0.067, 0.060, and 0.067, respectively). (**Table 2**)

**Table 2:** Comparison of depression, pain, cervical spine function and quality of life between the two groups.

	Control group (N = 85)	Intervention group (N = 32)	*P* value
BDI, score			
Preoperative	5.3 ± 2.1	12.6 ± 7.8	<0.001
Last follow-up	2.1 ± 1.5*	10.5 ± 7.2*	<0.001
Change	3.3 ± 1.9	2.4 ± 1.2	0.014
VAS, score			
Preoperative	3.4 ± 2.3	4.5 ± 2.1	0.020
Last follow-up	1.1 ± 0.9*	1.6 ± 1.0*	0.011
Change	2.2 ± 1.9	2.9 ± 2.1	0.087
JOA, score			
Preoperative	7.4 ± 3.6	5.6 ± 3.1	0.014
Last follow-up	15.1 ± 2.9*	11.9 ± 5.5*	<0.001
Change	7.6 ± 3.1	6.3 ± 3.2	0.067
NDI, %			
Preoperative	40.4 ± 10.2	48.2 ± 9.1	<0.001
Last follow-up	19.0 ± 9.1*	30.5 ± 10.3*	<0.001
Change	21.1 ± 11.4	16.8 ± 9.5	0.060
SF-36 PCS, score			
Preoperative PCS	35.3 ± 7.5	40.8 ± 9.8	0.002
Last follow-up PCS	27.6 ± 5.4*	33.3 ± 8.7*	<0.001
Change	7.4 ± 2.1	6.5 ± 2.9	0.067
SF-36 MCS, score			
Preoperative MCS	38.2 ± 8.3	44.2 ± 9.5	0.002
Last follow-up MCS	28.1 ± 7.2*	36.4 ± 8.4*	<0.001
Change	8.6 ± 4.3	7.1 ± 2.9	0.031

*Compared with preoperative, P < 0.05.

No significant differences were found in operative time (P=0.074), operative blood loss (P=0.060), hospital stay (P=0.083) and complications (P=0.542) between the two groups. (**Table 3**)

**Table 3:** Comparison of surgery-related outcomes between the two groups.

	Control group (N = 85)	Intervention group (N = 32)	*P* value
Operative time, min	78.4 ± 12.5	83.3 ± 14.6	0.074
Operative blood loss, ml	32.5 ± 5.1	34.8 ± 7.5	0.060
Hospital stay, d	5.8 ± 1.4	6.4 ± 2.2	0.083
Complications, n			0.542
PE/DVT	3	1	
UTI	7	3	
Pneumonia	5	2	

PE, pulmonary embolism; DVT, deep venous thrombosis; UTI, urinary tract Infection.

## Discussion

In this study, the preoperative BDI score of the intervention group was higher than that in the control group, suggesting that although depression patients received standard antidepressant therapy before surgery, taking anti-depression drugs could not control the symptoms of depression to the same level as normal people (16). However, after antidepressant therapy, the BDI scores of depression patients before ACDF were all at a low level, that was to say, most patients were in the so-called mild or moderate depression state (BDI score<15) before surgery, and almost none of patients with severe depression (17). This also indirectly supports the effectiveness of preoperative antidepressant therapy. Since the SF-36 MCS is a patient’s mental condition score, it has some similarities with the BDI score (depression score), that is, the preoperative SF-36 MCS of depression patients are higher than that of the normal group.

The preoperative clinical symptoms of cervical spondylosis patients complicated with depression were more obvious than those of the normal group, mainly reflected in high VAS score, low JOA score and high NDI score, which may be related to the higher preoperative BDI score in the intervention group and the more sensitive experience of uncomfortable feelings, such as pain and cervical dysfunction, in the depressed state (18, 19). This conclusion was similar to that of previous studies, that was, depression was an independent risk factor for obvious symptoms and poor treatment effect in cervical spondylosis patients (20). This was also the reason why we discussed whether depression is the contraindication of ACDF for cervical spondylosis.

In this study, the BDI score, VAS, JOA score, NDI, and SF-36 score were all significantly improved after ACDF, which means that cervical spondylosis patients with or without preoperative depression can all achieve good clinical outcomes from ACDF (21). There may be the following reasons: (1) The diseased intervertebral disc, marginal osteophyte and posterior longitudinal ligament were all removed during ACDF, which reduced the compression of spinal cord and nerve root, and thus provided good conditions for the recovery of neurological function (22); (2) ACDF alleviates neck pain and dysfunction which has a positive impact on the quality of life of patients (23); (3) ACDF was a mature surgical technique with short operative time and little operative blood loss, which greatly alleviates the pain of patients and has a positive impact on the psychological state of patients (24). The results showed that the long-term clinical efficacy of ACDF in patients with depression undergoing antidepressant treatment was similar to that in non-depression patients. This suggests that the previous view that depression is a contraindication of spinal surgery may need to be reassessed (7, 8). After antidepressant treatment, patients with depression can also obtain satisfactory long-term pain and life quality improvement from ACDF. Moreover, this result indicated that the relief of pain and disability after ACDF was also associated with the relief of depression (25). It was predicted that antidepressants after ACDF may also improve the relief of the pain and functional outcomes. Therefore, we don’t think depression is a contraindication for ACDF surgery, and preoperative and postoperative antidepressant therapy are helpful to relieve postoperative pain and improve life quality.

Antidepressants include selective serotonin reuptake inhibitors (SSRIs), serotonin norepinephrine reuptake inhibitors (SNRIs), tricyclic and tetracycline antidepressants, MAO inhibitors, 5-HT2 antagonists and so on (26). At present, studies have shown that SSRIs may result in abnormal bleeding in patients undergoing orthopedic surgery (13, 27). Mago et al. reported that the operative blood loss in patients taking SSRIs drugs was 2.5 times higher than that in the control group during lumbar fusion surgery (28), so it was suggested that preoperative platelet function test be conducted in patients with SSRIs taking, especially in elderly undergoing elective surgery, although there is no data indicating the benefits of this test (29). In this study, we found that the operative blood loss of patients taking antidepressants was similar to that of the control group. There may be the following reasons: (1) The baseline bleeding risk of patients included in our study maybe different from that of previous studies (30); (2) It was reported that, for complex spinal surgery, antidepressants may cause a significant increase in intraoperative bleeding, but a relative simple spinal surgery (such as ACDF) only had a small amount of blood loss (31); (3) There may be differences in the methods used in each study to record intraoperative blood loss (32); (4) In our study, we avoided using NSAIDs in patients taking SSRIs, which may reduce the risk of abnormal bleeding caused by SSRIs (33). In addition, there was no significant difference in the risk of postoperative complications related to platelet function, such as deep venous thrombosis and pulmonary embolism. These results confirmed that it was safe for patients with depression to use antidepressants before ACDF.

Our study has several limitations. First, we did not include some demographic and medical data (such as education level, platelet function test, etc.), which may affect the study results. Second, patients were not randomly divided. The decision of taking antidepressants and the type of antidepressants is entirely made by psychiatrists. Third, the duration of preoperative pain, depression and other symptoms could not be assessed, which may affect the results of the study.

Depression is not the contraindication of ACDF for cervical spondylosis. Depression patients who received preoperative antidepressants can achieve similar improvement of clinical symptoms from ACDF with non-depression patients.

## Data availability statement

The raw data supporting the conclusions of this article will be made available by the authors, without undue reservation.

## Ethics statement

The studies involving human participants were reviewed and approved by the Ethics Committee of the First Affiliated Hospital of Chongqing Medical University. The patients/participants provided their written informed consent to participate in this study.

## Author contributions

Conception and design: XD, YP, XC, and XL. Data analysis and interpretation: XD, YP, XC, YG, YL, and YT. Data collection and management: XD, XL, YF, HY, and KL. Manuscript writing and critical revisions: all authors. Overall responsibility: YP and XD. All authors have read and approved the manuscript.

## Conflict of interest

The authors declare that the research was conducted in the absence of any commercial or financial relationships that could be construed as a potential conflict of interest.

## Publisher’s note

All claims expressed in this article are solely those of the authors and do not necessarily represent those of their affiliated organizations, or those of the publisher, the editors and the reviewers. Any product that may be evaluated in this article, or claim that may be made by its manufacturer, is not guaranteed or endorsed by the publisher.
